# Redefining Prosthetic Needs: Insights from Individuals with Upper Limb Loss—A Systematic Review

**DOI:** 10.3390/s26020734

**Published:** 2026-01-22

**Authors:** Andreia Caldas, Demétrio Matos, Adam de Eyto, Nuno Martins

**Affiliations:** 1 ID+ Research Institute for Design, Media and Culture, School of Design, Polytechnic Institute of Cávado and Ave (IPCA), 4750-810 Barcelos, Portugal; dmatos@ipca.pt (D.M.); nmartins@ipca.pt (N.M.); 2 LSAD Research Institute, Limerick School of Art & Design, Technological University of the Shannon (TUS), V94 KX22 Limerick, Ireland; adam.deeyto@tus.ie

**Keywords:** upper-limb, amputation, user needs, prosthesis, wearable, requirements

## Abstract

Background: Upper limb loss has a profound impact on individuals’ daily activities, self-image, and social interactions. Despite continuous technological advances in upper-limb prosthetics, high rates of device abandonment persist, highlighting the need to better understand users’ functional and psychosocial needs. Methods: To gain a deeper understanding of the perspectives of upper limb amputees and the synthesis of their needs across ergonomic, functional, and psychological dimensions, this study was conducted. A systematic review was conducted following PRISMA guidelines to synthesize user-reported evidence on upper-limb prosthesis use. Articles indexed in the Web of Science database between 2016 and December 2023 were screened using predefined search terms related to upper-limb amputation, prostheses, social impact, and user needs. Studies were included if they reported direct perspectives of upper-limb prosthesis users regarding usability, functionality, and lived experience. Results: Out of 239 papers identified, 31 were included and analyzed. The findings reveal that functional performance, comfort, weight, intuitive control, and reliability are strongly interconnected with psychosocial factors such as confidence, embodiment, social participation, and acceptance. Technological advances have not consistently translated into improved alignment between prosthetic solutions and user needs, which is reflected in continued dissatisfaction and abandonment. Conclusions: This review provides a structured synthesis of user-reported needs across functional, ergonomic, and psychosocial dimensions, translating these insights into design-relevant guidelines. Emphasizing a user-centered and interdisciplinary perspective, the findings aim to support the development of upper-limb prosthetic devices that are more usable, acceptable, and aligned with users’ expectations, ultimately bridging the gap between user expectations and technological capabilities and promoting long-term adoption and quality of life.

## 1. Introduction

Worldwide, the number of amputations has been increasing. In 2017, an estimated 57.7 million individuals were living with limb loss due to trauma, which remains the leading global cause of amputation. The leading cause of amputation varies across regions; however, among traumatic causes, falls and road accidents are the most common, particularly among young men [1,2]. Amputations are typically categorized into two major groups, lower limb (LL) and upper limb (UL), each with distinct anatomical levels and functional implications [3,4,5]. Because of its central role in daily activities and social interaction, UL amputation poses unique challenges that extend beyond physical function. When an amputation occurs, numerous personal and social complications for the amputees are triggered [6,7,8]. Besides resulting in a decline in physical capacity also alters the patient’s pre-existing body image, leading to psychological limitations such as a break in self-esteem [6,9], and psychosocial challenges resulting in social barriers and isolation [5,10,11].

Prostheses, external devices that replace all or part of a missing limb, play a fundamental role in mitigating these consequences and supporting participation in activities of daily living (ADLs) [3,12,13]. Based on the global 2017 prevalence [2], more than 75,850 prosthetists would be required to provide adequate care for individuals with traumatic amputations.

Despite the technological advances in prosthetic design and control systems [12], high rates of abandonment and rejection persist, emphasizing the need to better understand the factors influencing long-term prosthesis acceptance [14]. With the increasing number of UL amputations, rising life expectancy, and limited user satisfaction [1,14,15], it is essential to analyze the specific needs expressed directly by prosthetic users. These needs span several domains, functional, ergonomic, and emotional/psychological, and strongly affect whether a prosthesis becomes truly embodied by the user.

The literature reveals a persistent gap between technical development and real-world acceptance, sustained use, and everyday integration of these devices by users. Existing studies tend to address isolated aspects of this challenge, such as the influence of early prosthetic fitting on neurodevelopment [16], control and cognitive load assessed through eye-tracking metrics [17], or technological evolution, materials, and research and development constraints [18]. Other reviews identify comfort and functional limitations as recurring reasons for device abandonment [19] and emphasize the importance of user-centered design, embodiment, and interdisciplinary collaboration [20,21,22], yet largely remain conceptual or technology-driven, without systematically synthesizing user-reported needs. Consequently, an integrative perspective that consolidates amputees’ viewpoints across ergonomic, functional, psychological, and social dimensions is still lacking.

This systematic review addresses this gap by synthesizing recent user-centered evidence to derive actionable guidelines for the development of prosthetic devices that are more usable, acceptable, and aligned with users’ expectations. In this context, usability-related factors such as comfort, weight, control, and reliability are closely coupled with psychosocial outcomes, as difficulties in functional performance often lead to frustration, reduced confidence, and social withdrawal, whereas improvements in intuitive use and comfort support embodiment, confidence, and social participation. Understanding this interdependence is essential to reducing abandonment and promoting long-term prosthesis acceptance.

Our goal is to provide clinicians, researchers, designers, developers, and end users with an updated overview of the state of the art, thus encouraging interdisciplinary efforts to address challenges related to prosthetic use, organized in three conceptual groups: Adaptation/Usability, Functionality/Performance, and Psychological Impact.

This article is structured as follows: Section 1 presents the Introduction; Section 2 provides the Background and fundamental concepts; Section 3 describes the Methods; Section 4 presents the Results; Section 5 offers a Discussion and design guidelines; and Section 6 provides the Conclusion.

## 2. Background

**Figure 1:**
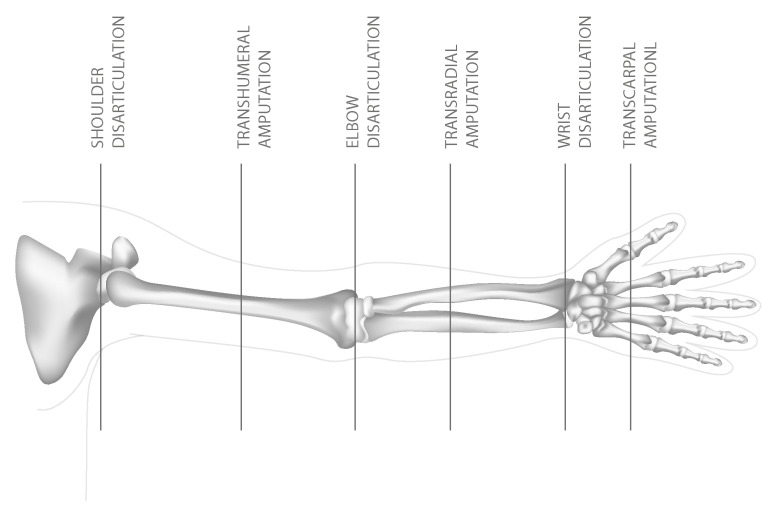
Levels of upper-limb amputation.

The human hand is a highly sophisticated structure with a fundamental role in everyday function, environmental interaction, and social communication [5,13,23]. Given its complexity, upper-limb amputation significantly impacts users’ independence and quality of life. UL amputation encompasses different anatomical levels (Figure 1), including Shoulder Disarticulation (SD), Transhumeral (TH) amputation, Elbow Disarticulation (ED), Transradial (TR) amputation, Wrist Disarticulation (WD), and Transcarpal (TC).

### 2.1. Anatomy and Function of the Human Hand

**Figure 2:**
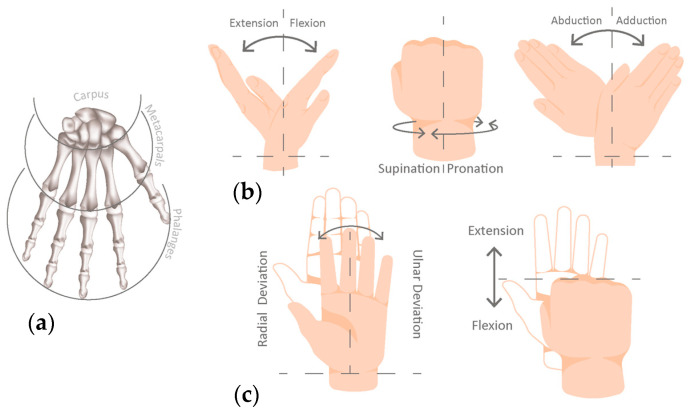
Human hand anatomy (**a**) skeletal anatomy of the hand; (**b**) Wrist motions; (**c**) Radial and ulnar deviation of the wrist, along with finger flexion/extension. Dashed lines indicate the anatomical neutral reference position, while arrows represent the direction of movement.

The human hand performs three fundamental functions: grasping, manipulating objects, and exploring the surrounding environment, enabling refined physical and sensory interaction [24]. Structurally, it consists of 19 bones together with muscles, ligaments, tendons, blood vessels, nerves, and soft tissues. Intrinsic muscles predominantly facilitate flexion and extension at interphalangeal joints and radial–ulnar deviation (Figure 2c). The thumb, with its unique anatomical configuration, contributes significantly to manual dexterity due to its increased Degrees of Freedom (DoFs) [25].

Anatomically, a normal human wrist-hand structure can be divided into three main regions, carpus, metacarpus, and phalanges (Figure 2a), and presents a total of 27 DoF. Of these, 21 DoFs pertain to finger joints are essential for performing various grasping patterns crucial in ADLs, and the remaining 6 DoFs on the wrist [5,26,27].

The arrangement and individual mobility of the eight carpal bones enhance wrist motion, allowing extension (≈70°), flexion (≈75°), adduction (≈35°), abduction (≈20°), and forearm supination–pronation (0°–180°) (Figure 2b) [13,25]. The hand weight range is between 283 g and 565 g, and the forearm weight range is from 755 g to 1400 g [28].

### 2.2. Prostheses Classification

Upper-limb prostheses are commonly categorized according to the amputation level: Transhumeral (TH), Transradial (TR), Transcarpal (TC), and Transmetacarpal amputations [28].

Prosthetic devices can be divided into passive and active categories. Passive, or cosmetic, prostheses offer limited functional capability and are mainly used for aesthetic purposes, helping to reduce social discomfort [4,5,29]. Active prostheses restore part of the lost motor function through mechanical or electrical force transmission [28,30]. Mechanical or body-powered (BP) prostheses operate through body movements transmitted via harnesses or cables and are typically preferred for their robustness, low maintenance, and suitability for heavy-duty tasks [4,12,13,28,31].

Electrical prostheses rely on external controllers, buttons, electrodes, or sensors to activate motors responsible for movement. These systems require battery power to operate and enable programmable control strategies [28]. Among them, myoelectric (MYO) prostheses, which detect EMG signals from residual muscle contraction, represent the most prevalent control method in UL prosthetics [4,20,28,31].

Even with advances in prosthetic technology [12], device abandonment remains a significant issue [14]. Understanding user needs, including functional performance, ergonomics, and emotional/psychological integration, is therefore crucial for improving device acceptance and long-term use.

## 3. Methods

### 3.1. Search Strategy

**Figure 3:**
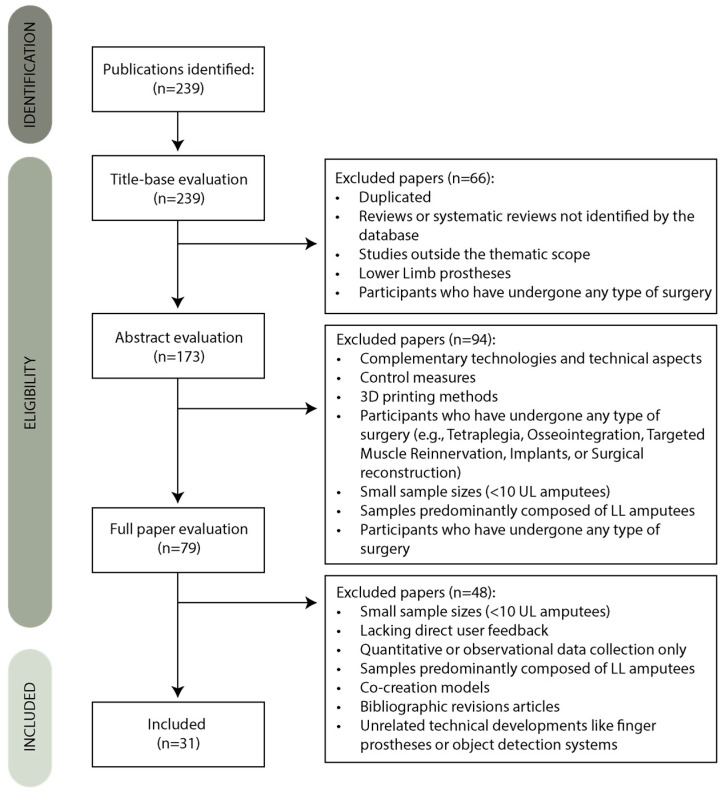
Systematic review flowchart.

In this section, we describe the methodology employed to conduct the present systematic review, providing an overview of the strategy and criteria used to select relevant papers (Figure 3). A literature review was performed to identify the most relevant quantitative and qualitative studies following the Preferred Reporting Items for Systematic Reviews (PRISMA) guidelines.

To define the timeframe for inclusion, a preliminary search for systematic reviews and reviews was conducted in the Web of Science database using the following keywords: (ALL = Transradial amputation OR ALL = upper limb) AND (ALL = prosthesis OR ALL = prosthetic) AND (ALL = social impact AND ALL = patient needs). As a reference, the work by F. Cordella et al. [5] provides a systematic review of the studies up to that date and is considered the starting point for this research. In this sense, this systematic literature search was then performed in the Web of Science database, covering studies published from 2016 to December 2023 using the defined search terms. The use of the Web of Science database influences the type of literature captured, favoring studies that explicitly link prosthesis use, user experience, and design-related outcomes, in alignment with the objectives of the present review.

### 3.2. Selection Criteria

From the articles retrieved using the search strategy described above, a set of criteria was established to identify the studies eligible for this review. As an initial step, review articles were excluded automatically.

For this systematic review, the following inclusion criteria were applied to identify relevant publications:

- Research focused on UL prosthesis users, addressing aspects such as comfort, utility, and appearance.
- Studies involving users of both passive and active prostheses.
- Research detailing user preferences regarding specific features, priorities for prosthetic design, and tasks users aim to perform in daily life with the prosthesis.

The articles meeting the selection criteria underwent detailed review and analysis.

### 3.3. Search Results

The search in the database, using the strategy previously presented, resulted in 239 publications. Initially, 160 articles were excluded: 66 based on title and 94 based on abstract relevance. Among these, one duplicate and six reviews or systematic reviews not identified by the database were excluded. Additional exclusions were made for studies outside the thematic scope, such as those focusing on complementary technologies and technical aspects like control measures, unmet needs, three-dimensional (3D) printing methods, simulator development, or protocol analysis (*n* = 51). Studies focusing on LL prostheses or involving participants with a specific condition in addition to amputation or participants who have undergone any type of surgery (e.g., Tetraplegia, Osseointegration, Targeted Muscle Reinnervation, Implants, or Surgical reconstruction) were also excluded (*n* = 34) as these conditions and interventions introduce additional functional, clinical, and technological factors that may significantly influence user needs, expectations, and prosthesis interaction. Such factors can provide users with access to capabilities, control strategies, or rehabilitation pathways that are not available to individuals using conventional options. Further exclusions were made in subsequent phases for articles with small sample sizes (<10 UL amputees) (*n* = 17), as very small samples limit the robustness and generalizability of the findings and reduce the reliability of user-need synthesis, or samples predominantly composed of LL amputees (*n* = 44). Articles lacking direct user feedback or failing to analyze such feedback were excluded as well (*n* = 7). A full-text review led to the exclusion of an additional 48 articles. These included studies with fewer than 10 UL amputees in the sample (*n* = 28), samples predominantly composed of LL amputees (*n* = 2), and participants with unrelated conditions (*n* = 2). Articles without direct amputee feedback or where such feedback was not analyzed were also excluded (*n* = 7), as were those focusing on quantitative or observational data collection only (*n* = 2). Lastly, articles addressing topics unrelated to UL prosthesis development, such as co-creation models, bibliographic revision articles, or unrelated technical developments like finger prostheses or object detection systems, were excluded, and one was inaccessible. The last 31 articles match the defined criteria and will be discussed in this work.

Although a large number of publications were initially identified, the final selection of 31 studies reflects a deliberate methodological choice. This review prioritizes studies that provide direct user-reported evidence on prosthesis use, rather than broader technical, clinical, or conceptual discussions. By applying strict inclusion criteria, the review ensures analytical depth and coherence in the synthesis of functional, ergonomic, and psychosocial needs, allowing for a focused interpretation of challenges directly experienced by upper-limb prosthesis users.

## 4. Results

**Figure 4:**
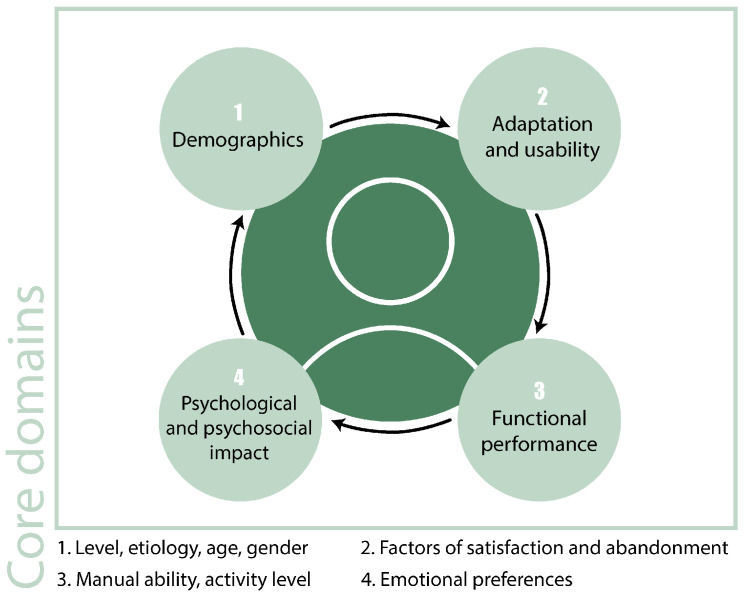
The figure overview of the groups’ needs throughout our review, as well as the relationship between them.

This chapter provides an integrated synthesis of the key findings documented in the literature addressing the use of upper-limb prostheses. Organized into four primary domains (Figure 4), participant demographics, adaptation and usability, functional performance, and psychological and psychosocial impact. The organization presented in Figure 4 is used as an analytical framework to structure and synthesize findings across the reviewed studies. This framework was derived inductively from recurring themes consistently identified in the literature and reflects how demographic factors, adaptation and usability, functional performance, and psychological and psychosocial impact are interrelated in users’ experiences of prosthesis use. It is not intended as a comprehensive or predictive model of prosthetic development, but rather as a conceptual tool to support the integrated interpretation of heterogeneous user-reported evidence.

These domains are closely related, even if they are studied independently. Users’ functional needs as well as their aesthetic and emotional preferences are directly shaped by demographic factors such as age, gender, amputation level and etiology, and type of prosthesis used. The adoption of prosthetic devices is influenced by aspects such as comfort, weight, durability, and clinical assistance. These factors also contribute to the explanation of patterns of satisfaction and abandonment.

### 4.1. Demographics

**Figure 5:**
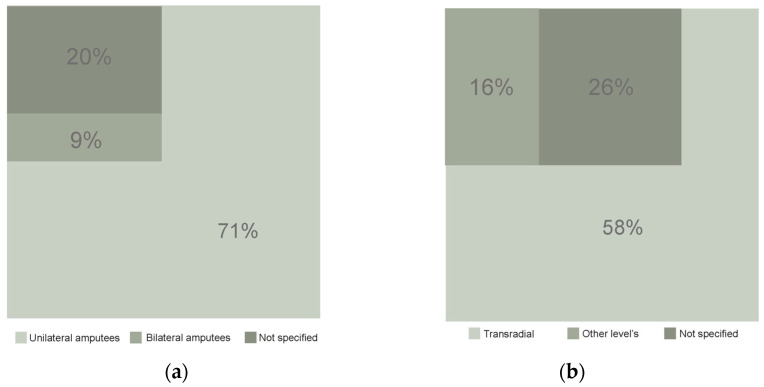
(**a**) Overview of the types of amputation represented in the participant samples across the articles included in the review, indicating the relative frequency of each amputation type reported in the analyzed studies. (**b**) Overview of the amputation levels most frequently reported in the included articles, highlighting the anatomical levels predominantly addressed in the literature.

All studies included in this review contributed to the demographic analysis. Of the total number of articles included in this review, the number of participants (considering only amputees when indicated) is 4885 individuals. Of these, 3468 (70.99%) have unilateral amputation, 431 (8.82%) have bilateral amputation, while the remaining participants either did not specify the type of amputation or had other types of amputation (Figure 5a). The sample of the included studies is mostly composed of men. Five studies do not report the gender composition of the sample. Among those that do, four studies indicate that their samples are predominantly composed of women, representing a total of 55 female participants, and one study reports an equal distribution of men and women in the sample. Regarding the level of amputation (Figure 5b), TR amputation (below-elbow, including WD) was identified as the most prevalent in all the articles reviewed (58%). In 25.8% of the studies, the level of amputation was not specified, and 16.12% reported samples predominantly composed of amputees with other levels of amputation, such as TH (above-elbow) amputations or SD. In total, 1591 participants had TR amputation.

**Figure 6:**
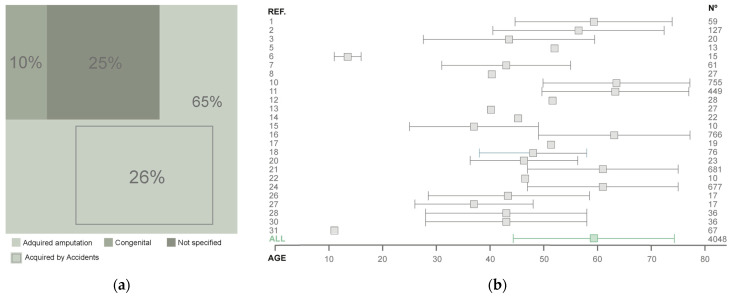
(**a**) Overview of the primary causes of amputation reported in the participant samples of the included articles, summarizing the most common etiologies represented in the reviewed studies. (**b**) Mean age of participants reported in the 27 articles for which age data were available, providing a general characterization of the demographic profile of the included study samples.

From an etiological perspective (Figure 6a), among the articles included in this review, 65% indicate that amputation predominantly occurs due to acquired (traumatic) causes, with the most common cause being accidents, representing the most prevalent cause in 26% of the studies reviewed. Regarding age, data on participants’ ages were retrieved. The weighted average for the entire sample used in the included articles shows that the mean age of the 4048 participants is approximately 59.33 ± 15.08 years (Figure 6b).

In relation to the type of prostheses used by participants, nine studies do not provide this information. Among the studies that do report prosthesis categorization, two indicate that the majority of participants use aesthetic prostheses. In eleven studies, the sample predominantly consists of users of MYO/electric prostheses. Finally, nine studies report that their samples are primarily composed of users of mechanical or BP prostheses.

### 4.2. Adaptation and Usability

The works included in this chapter address the needs and challenges faced by prosthesis users, focusing on satisfaction and the causes of prosthesis abandonment. This enables the identification of common trends and user preferences.

The use and acceptance of prostheses are complex topics, influenced by factors that either contribute to user satisfaction or lead to abandonment. Reasons for abandonment are often linked to practical and physical difficulties, while factors that promote satisfaction are commonly associated with features that meet not only practical but also emotional needs [32,33,34,35,36,37]. For instance, L. Resnik et al. [34] state that no significant differences were found in satisfaction scores among different types of prostheses, except in specific cases related to amputation level [38] and participant age, although the sample composition limits generalization.

Some studies indicate that prosthesis choice and use also differ by level of amputation, gender, and age group [36]. For example, individuals with more proximal (higher) amputations often express a preference for prostheses that maximize functionality and control, such as MYO devices, while those with more distal (lower) amputations may prioritize lighter and easier-to-use mechanical prostheses [34]. Women tend to favor aesthetic and multifunctional devices, while men show a greater inclination toward mechanical prostheses. Similarly, children favor aesthetically distinct prostheses, such as those inspired by superheroes [39], whereas older users often report a preference for prostheses with a more natural appearance [32,38,40]. Older users and those spending more time at home demonstrate less concern with weight and dexterity [37] compared to younger users, who prioritize lightweight and usability features due to their active lifestyles, including work and recreational activities [40,41].

Prosthetic aesthetics are reported to influence user acceptance and social impact. Many amputees report a preference for devices that resemble natural limbs [42], citing the goal of going unnoticed. Conversely, others, particularly children, opt for unique designs that reflect their personality and identity [32,33,39,41,43]. Social perceptions and interactions are also noted as relevant, as some users experience discomfort when wearing prostheses in public [30,41].

Active prostheses that are user-friendly and suitable for daily and leisure activities are consistently identified as important. Functionality is frequently cited as a key factor, as devices that facilitate day-to-day tasks tend to receive positive evaluations in the literature [32,34,35,44]. However, in the Korean context [32], users report a preference for cosmetic prostheses, even though most studies identify functionality as a fundamental criterion for acceptance [32,33,35,37,40].

Weight and comfort emerge as other critical criteria influencing prosthesis use, particularly in functional devices, which are often heavier than passive ones [36]. Excessive weight is associated with discomfort and fatigue during daily use, prompting users to opt for lighter devices or to abandon use altogether [33,34,35,36,37,38,40,41]. Discomfort is often linked to poor prosthesis fit and lack of personalization, causing issues such as friction, skin irritation, and pain during prolonged use [33,39,44]. These factors contribute to a described demand for lighter and more comfortable prostheses, which has driven interest in promising technologies such as 3D printing and soft robotics. These technologies not only enable to address some of the needs mentioned, allowing the creation of personalized and lightweight devices, but also are being investigated for their potential to make prosthetic solutions more accessible, particularly in low- and middle-income countries [35,37,38,39,40]. However, these studies emphasize that technology alone does not address all issues. A user-centered approach and co-creation methods are widely recognized as necessary to ensure that prostheses effectively meet user needs and preferences. User-centered design and co-creation methods [30,39,40,45] involving prosthesis users, engineers, and designers are proposed as methods to ensure devices address both functional requirements and users’ emotional and social concerns. Jones et al. [45], for example, report benefits from conducting research co-creation outside controlled environments (e.g., laboratories and clinics). Users are willing to participate in remote research, provided they have control over data sharing and effective communication with researchers. This integrative approach has been associated with the development of more adaptable and acceptable devices, and with improvements in quality of life for users.

Beyond weight and comfort, durability is another critical criterion [33,34,35,38,39]. Prostheses requiring frequent repairs are less commonly used or reported as satisfactory. Some devices, particularly MYO ones, are described to be fragile, increasing maintenance costs and limiting continuous use [33]. Mechanical prosthesis users, however, frequently report prioritizing durability, even if it entails slightly more weight [34]. The literature indicates that balancing lightweight design and durability, along with faster repair processes, is a key factor for improving prosthesis acceptance [33,34,38,39,41].

As the price frequently impact the functionality that consequently impacts the usability, is another frequently mentioned factor [33,35,37,38] included articles in this section. Also, it is stated to influence prosthesis selection, highlighting affordability as a factor in development. There is also a reported preference for prostheses offering greater quality (greater dexterity and control), with proposals in the literature for developing more intuitive interfaces and sensory feedback capabilities. Such features are hypothesized to enable users to achieve more precise movement control [30,33], which will be explored further in the next topic (Functionality and Performance).

For congenital amputees, Walker et al. [33] reveal that many adapt to their condition without prostheses, suggesting that prosthesis use is not a universal necessity. Acceptance and social and professional impacts are reported to be less pronounced for these individuals compared to those who became amputees due to accidents. Johansen et al. [44] indicated that it is different for children with congenital unilateral amputation; they find the prostheses practical and use them for many kinds of activities.

Although not directly related to prosthesis development, the lack of training and psychological support can act as a barrier to prosthesis acceptance and use [30,36,44]. Users often face adaptation challenges due to inadequate support, indicating a need for more personalized clinical services and accessible training programs. Psychological support is also identified as important, as emotional adaptation to prosthesis use is an essential aspect of improving users’ quality of life.

In summary, balancing comfort, functionality, and desired aesthetics, which vary from user to user, is determined factor for satisfaction and abandonment. This demonstrates the relevance of a user-centered approach focused on real needs and preferences [33,37,40].

### 4.3. Functionality and Performance

This chapter, focused on the functionality and performance of prostheses aims to identify the benefits and limitations that contribute to the optimal functionality and performance of prostheses, which in turn influence user satisfaction and usage.

Analyzing the results revealed that the level of amputation was significantly associated with functional performance, as was the type of prosthesis used [43,46,47,48,49]. Resnik et al. [46], which focuses on performance in daily activities and dexterity (defined as the speed or responsiveness of prosthetic movements, grip options, terminal device design, and the need to switch between grips or movements to guide the terminal device during activities), showed that dexterity and performance in ADLs varied significantly by amputation level. Distal amputations, such as TR, showed better outcomes, while proximal amputations demonstrated worse results [43,49]. Non-users reported a greater need for assistance in performing daily living tasks, supporting the role of active prostheses in improving quality of life [50]. However, regarding ADLs performance, one article indicated no significant differences in task completion difficulty with or without a prosthesis. This contrasts with the authors L. J. Resnik et al. [46,49], which found that non-users had poorer performance in dexterity tests, with the least limitations observed in writing tasks and the greatest difficulties in tying knots or bows and cutting food with utensils [49].

Despite the amputation level and prosthesis use, daily tasks are challenging for all amputees. L. J. Resnik et al. [49] reported that tasks requiring two hands, such as passing a 20-pound turkey or ham, were difficult regardless of amputation level or prosthesis use, although non-users found these tasks even more challenging. Tasks such as washing one’s back, changing a light bulb, buttoning a shirt, and cutting paper with scissors were reportedly harder for prosthesis users than for non-users. Among amputees, those with TH or SD amputations faced more difficulty in tasks like passing heavy objects compared to TR amputees, who, in turn, struggled more with buttoning shirts and washing their backs. Users of cosmetic prostheses, which improve body image and psychosocial adjustment, stated greater difficulty in performing ADLs compared to users of BP and single-function (single-DOF) MYO prostheses. BP prostheses performed better in areas such as lifting bulky objects and household chores, with these tasks deemed easier compared to other prosthesis types [50]. L. J. Resnik et al. [46] found that users of BP devices achieved better dexterity in manipulating small objects than users of MYO prostheses. However, differences between prosthesis types were not statistically significant regarding health-related quality of life (HRQOL) or community integration. Similarly, L. J. Resnik et al. [50] reported no significant differences between BP, single-DOF MYO, and multi-DOF MYO prostheses regarding activity difficulty, need for assistance, self-reported disability, or HRQOL.

Data from L. J. Resnik et al. [46] indicate that having a more expensive prosthesis, such as a multi-DOF MYO device, does not necessarily provide more advantages. Kannenberg et al. [47] reported consistent findings, comparing two multi-articulated MYOprostheses e.g., i-limb (Össur, Iceland) and bebionic (Ottobock, Germany) and showing that a greater variety of grip types did not translate into greater ease or utility compared to the Michelangelo (Otto Bock HealthCare GmbH, Duderstadt, Germany) model, which has fewer grip options. Difficulty in intuitively activating different modes and the time required to switch between functions was described to lead users to rely on their intact hand or compensatory mechanisms rather than fully utilizing the prosthesis.

Focusing on bilateral amputees, Sears et al. [48] compared BP and electric prostheses (Electric Hooks and Electric Hands, including a multi-articulated model). The study found that electric hooks performed slightly better in the number of tasks completed and grip security, potentially due to greater grip strength and speed. However, as in L. J. Resnik et al. [46], these differences were not statistically significant. When comparing hooks specifically (mechanical vs. electric), electric hooks consistently performed better across all measures. Nonetheless, mechanical hooks were selected for noise tolerance, comfort, and cost. Despite this, active prostheses were reported to be especially useful for task requiring fine motor skills [50]. Even though a greater variety of grip types did not translate into ease of use or utility, Davidson et al. [51] indicated that adding more DOFs to a device could increase complexity, making usage more challenging. However, participants expressed a willingness to learn to manage more complex control systems if these offered greater functionality. Davidson et al. [51] also examined limitations of current prosthetic devices fail to meet users’ needs, particularly regarding control limitations that prevent full articulation of prosthetic wrists. Participants identified Dart Thrower’s Motion (DTM), a combination of elbow flexion/extension, shoulder rotation, and wrist movement, as critical for daily activities, with lower satisfaction described when prosthetic devices could not accommodate this motion. When asked about desired improvements, most participants identified as priorities individual finger control, a movable thumb, stronger grip strength, and faster movements. In contrast to Davidson et al. [51], L. J. Resnik et al. [49], which evaluated the psychometric properties of three versions of the Patient-Reported Outcomes Measurement Information System (PROMIS-EU) system’s upper extremity short forms (6, 7, and 13-item versions), adapted to include tasks requiring both hands, showed lower satisfaction with prosthetic devices for DTM-related activities compared to other tasks. Zhang et al. [43] evaluated the relationship between perceived function (user-reported) and actual function (measured by performance tests). While the sample size was insufficient for definitive conclusions, results indicated a lack of significant association between perceived and actual function, identifying a need for further research.

While advanced technologies such as gesture control and pattern recognition have the potential to enhance usability, their impact on user experience remains uncertain. Kannenberg et al. [47] stated that patients often prioritize natural appearance over speed or ease of use in prostheses, sometimes compensating for increased time to perform tasks.

Regarding control, Touillet et al. [52] investigated phantom limb movements (PLM) as a control method for MYO prostheses with multiple active joints. Thirteen types of PLM were identified, with most patients able to perform several types. Although PLM tended to be slower and smaller in amplitude compared to intact limb movements, training improved speed and endurance, demonstrating feasibility for prosthesis control. Factors such as amputation level, time since amputation, chronic pain, and prosthesis use did not appear to affect PLM.

In sensory feedback, Jabban et al. [53] identified its role in user confidence, particularly in activities like holding objects or shaking hands. Reliable sensory feedback was identified as crucial, complementing user strategies such as visual cues or vibrations through the socket. Participants reported a need for feedback to cover various hand areas and proposed that lightweight, customizable, and silent systems could enhance trust and improve functionality.

Studies on the Deka Arm (Manchester, NH, USA) [54,55,56,57] reported significant findings. L. Resnik et al. [54] stated improved dexterity and activity performance after initial training, though daily usage decreased over time. L. Resnik et al. [55] found that most participants could perform new activities with the Deka Arm, although some tasks, like yard work or using powered equipment, were exclusive to their personal prostheses. L. Resnik et al. [56] and L. J. Resnik et al. [57] compared control systems, finding that inertial measurement units (IMU) outperformed MYO control, Electromyography Pattern Recognition (EMG-PR) in dexterity and user satisfaction, despite reports of increased pain from IMU usage.

In summary, the most common issues included difficulties with daily tasks, grip security, discomfort, and the need for prosthetic reliability. Across the reviewed studies, users described unmet functional needs across different prosthesis types. In response to these limitations, some users described alternating between different prostheses depending on the task or context, with perceived differences in satisfaction and functionality across devices.

### 4.4. Psychological and Psychosocial Impact

This chapter aims to understand the psychosocial impact of prosthesis use among amputees, the benefits provided in social interaction, as well as the barriers to social reintegration and strategies employed to overcome them. The goal is to identify common obstacles to guide the development of prostheses that improve amputees’ quality of life by addressing aspects such as users’ self-perception and meta-perception (how they believe others view them), factors that are crucial for the acceptance and social integration of prostheses mentioned previously [58]. The literature [42,58,59,60] examines the psychosocial impact of prostheses and their influence on users’ social reintegration, reporting that prostheses can improve users’ self-image and social perception. These studies document the role of prostheses in social interaction and the reintegration of amputees. Kristjansdottir et al. [42], which focuses on the use of cosmetic prostheses, describe that these devices are considered essential for social adaptation and interaction, particularly because they help “normalize” users’ appearance, facilitating interactions and providing the confidence necessary to return to work. This need for social acceptance is also reported by Bretschneider et al. [58], which describes how prostheses change how users are perceived by others, increasing acceptance and reducing pity and stigma. This is true both in more developed contexts [58] and in cultural settings with additional challenges [60]. In both environments, despite cultural differences, discrimination experiences, and the need for social support, they are reported to influence the psychosocial impact of prostheses. Regardless of the type of prosthesis, they are associated with reducing stigma and facilitating social acceptance, not only through technical capabilities but also by reducing visual differences [58,59,60]. Hutchison et al. [59], which explores barriers and facilitators of community reintegration, complements this perspective by showing that prostheses are critical for participation in social activities and indispensable for integration in community settings. Similarly, participants in Ramirez et al. [60], who focused on experiences in Uganda, described prostheses as crucial for adaptation and social acceptance, reporting a need for a socially recognized identity.

Despite these benefits, the studies also report challenges and limitations associated with using prostheses in social contexts. L. J. Resnik et al. [61] identify mental effort and focus required to handle prostheses, limiting their use in ADLs, particularly in social situations (e.g., handshakes or hugs with strangers). In Uganda [60], participants also reported difficulties performing everyday tasks, leading many to rely on residual limbs or family assistance, reflecting functional challenges similar to those identified in other studies [59,61]. These papers indicate that, for some individuals, prostheses may feel burdensome, depending on the context and personal acceptance.

The psychosocial impact of prostheses is reflected in studies that examine the link between prosthesis use and users’ identity. Bretschneider et al. [58] reports that bionic prostheses boost self-esteem and confidence, enabling users to feel more independent and less reliant on help. Similar feelings of reduced social stigma and greater self-confidence are described in Hutchison et al. [59], where participants noted that prostheses help reduce the visibility of their disability and facilitate inclusion. L. J. Resnik et al. [61] explore the psychosocial impact and reveal that many users experience anxiety and insecurity in public settings, particularly when not wearing their prostheses, while Ramirez et al. [60] discuss feelings of inadequacy and experiences of social discrimination. Participants in these studies employed various strategies to cope with social stigma.

Finally, social acceptance and professional reintegration emerge as recurring themes. Kristjansdottir et al. [42] reports that cosmetic prostheses are described as supporting return to work, where a normalized appearance facilitates interaction and integration. In Bretschneider et al. [58], users of bionic prostheses described positive acceptance in professional environments, describing respect and equality in their interactions with colleagues and supervisors. Hutchison et al. [59] also documents the importance of prostheses for social acceptance at work, noting that by enabling tasks requiring two hands, prostheses promote participation in leisure activities, thereby contributing to inclusion.

Prostheses are associated with changes in users’ self-perception and experiences in social contexts and influence community participation by shaping how individuals are perceived by others [42,59,60].

## 5. Discussion

The discussion integrates both quantitative and qualitative perspectives: quantitative trends are derived from the number of studies and sample sizes associated with each need (Table 1), while qualitative insights are drawn from the interpretation of users’ experiences, perceptions, and reported challenges described in the literature.

In developing this review, the included studies were organized into four conceptual groups reflecting the dominant themes consistently reported in the literature: demographics, adaptation and usability, functionality and performance, and psychological and psychosocial impact. These categories were selected because they emerge across major studies as the primary determinants of prosthesis satisfaction, acceptance, and long-term use. Across the literature, it also became evident that user needs are deeply interrelated and multifaceted. Even when individual studies focus on a specific domain, their findings intersect with broader issues. For this reason, the identified needs were synthesized into four overarching categories: Ergonomic Needs, Functional Needs, Psychological Needs, and Other Needs, allowing a clearer representation of recurring patterns while acknowledging their interdependence. For each category, relevant references and estimated sample sizes were identified to contextualize the strength and representativeness of the evidence, with sample size values reflecting the cumulative number of participants across studies reporting each specific need, acknowledging that individual samples may contribute to multiple categories.

The Discussion is organized around the main user needs: Ergonomic Needs, Functional Needs, Psychological Needs, and Other Needs; and their respective sub-domains, identified from user-reported needs and summarized in Table 1, which collectively structure the analysis presented below. The Functional Needs correspond to the physical activities users wish to perform and the requirements necessary to execute them. Psychological Needs represent the mental and cognitive demands expressed by amputees regarding the use of the device, both in personal contexts and social interactions. Additionally, participants highlighted needs related to access to clinical services. Although these needs are not directly linked to prosthesis development, they were consistently stated as having a significant impact on users’ psychological experience, particularly by reducing anxiety, increasing confidence, and supporting emotional adaptation during prosthesis use. The Ergonomic Needs pertain to user-identified requirements concerning the interaction between the residual limb and the prosthesis, focusing on reducing discomfort or the risk of injury while promoting consistent device use. Finally, the Other Needs category includes user-identified requirements that do not fit into the aforementioned categories (see Table 1).

In this regard, the table indicates a comprehensive collection of needs of UL prosthesis users that were not fully addressed with commercial prostheses. Converting these user needs into fundamental design guidelines is essential for developing user-centered UL prosthetic devices for future work.

Studies suggest that cosmetic prostheses are most often used during the initial phase of adaptation to prosthesis use. These are generally the first options adopted after amputation, serving as a means of adjusting to the new body image with the purpose of concealing the injury and normalizing appearance [33,37,42]. Initially, they play a vital role in facilitating the return to life, both in private and social contexts. However, over time, they tend to be abandoned as the individual and those around them become accustomed to the new appearance, and the condition no longer attracts attention [33,42]. Notably, Kim et al. [37] emphasize that this choice varies according to the personal needs of the user. Nonetheless, the need for functionality is evident in the analyzed studies. Active prostheses are generally the most adopted, as they allow amputees to regain some of the functionalities lost due to amputation [33,36,43,58]. However, prostheses are predominantly perceived as tools rather than as extensions of the self [32,38,45,53]. In addition to the perceived non-need for a prosthesis, it is evident that prosthetic design is still far from ideal [32,37,53]. This may be attributed to the fact that studies on prostheses are primarily focused on technical aspects [37]. Therefore, it is essential to understand the reasons behind these shortcomings and identify opportunities for improving current devices.

One of the primary roles of a hand is to grasp and manipulate objects [37]. Thus, functional needs are among the most prominent requirements of patients, with functional dissatisfaction frequently reported [30,32,33,41,42,43,47,50,53]. In this context, difficulty in control is widely noted [32,41,53], alongside an explicit desire for more intuitive systems [36,47,53,61] that are easy to learn and operate [35,41,51] and reduce the mental effort required for their use [36,40,53]. This, in turn, increases user confidence in the device [42,45,58,59,61]. The goal is to enable users to focus on activities rather than being preoccupied with the device itself [53]. These requirements underscore the importance of aligning prosthetic movements with the functions of the required types of prehension (grasp and hold) [33,37,38,40,46,47,48]. Functional development must address these needs, possibly through solutions that help reduce compensatory movements [41,43,45,53] and allow the contralateral arm to remain free [41]. Key requirements include the incorporation of a movable thumb [38,48,51] and the independent actuation of flexible fingers [38,41,47], which are essential for facilitating natural movements such as global hand opening/closing, a strong, fast, and reliable pinch grip opening/closing [38,41,47,48,50,52,53], and flexion/extension and abduction/adduction of the fingers [37,51,52]. Additionally, wrist functionalities, such as flexion and extension, radial and ulnar deviation, and motorized pronation/supination [33,35,47,48,51,52,54], are frequently cited as desirable improvements that could enable users to lead more independent lives. It is equally crucial to align these advancements with the prosthesis’s functional dexterity, prioritizing its ability to perform ADLs, such as personal care, household tasks [33,35,39,40,45,50], work or school tasks for children [37,40,42,44,58], and leisure or sports activities [40,45,55,59,60]. The focus should be on natural and efficient solutions, avoiding overly complex systems, which do not always lead to higher user acceptance [30,34,39,46]. However, Davidson et al. [51] indicate that some participants are willing to accept more complex systems if they offer greater functionality.

Regarding the evaluation of functionality and performance, the analysis of the literature highlights the absence of a single, universally standardized method for assessing the performance of upper-limb prostheses. Instead, studies consistently rely on a set of complementary approaches that combine objective and subjective measures. The most commonly used include the assessment of ADLs, manual dexterity tests and task-based functional performance evaluations, as well as patient-reported outcome measures, such as the PROMIS. Some studies also integrate comparisons between perceived function and function objectively measured through performance tests, revealing relevant discrepancies between users’ subjective experience and their actual performance. Taken together, these approaches constitute the most transversal framework for functional performance evaluation identified in the literature, enabling a more comprehensive characterization of prosthetic effectiveness, despite the absence of a universally accepted evaluation protocol.

Battery life and operating time are additional needs highlighted by participants in connection with functionality. While some consider the autonomy inadequate due to high energy consumption, others find it sufficient, with many expressing a desire for the ability to independently replace the battery [33,35,38,56].

Functionality not only promotes user independence [38,40,58,60] but is also directly linked to social integration [46,53,59,61]. Like the human hand, prostheses serve as facilitators of communication and social interaction [30,42,53,58,60], fostering a sense of security and social inclusion while reducing the stigma associated with amputation [42,58,60]. However, users report difficulties with acceptance, mentioning feelings of distress, rejection, and discrimination [58]. The lack of fine motor control [33,35,38,58] and sensory feedback [33,47,48,53] are identified as barriers that, if addressed, could drive significant advancements in this regard. Developing prostheses with control systems that enable delicate interactions, such as handshakes [39,53,61], dining out (e.g., using cutlery and holding glasses) [45,50,59,61], or caring for a baby [38,60], is widely desired. These activities require natural, intuitive, and flexible movements, with smooth transitions between different grasp patterns [39,46,47,58]. Combining fine motor control with sensory feedback, whether tactile, vibratory, or pressure-based [35,38,48,59] can help achieve these goals. Sensory feedback that is customizable and adjustable for each user, provided through a reliable, lightweight, and silent system, is seen as a promising solution. Not only does it enhance the user experience, but it can also improve proprioception and confidence in using the device [33,38,48,53]. However, it is important to consider the findings of Einfeldt et al. [38], which suggest that sensory feedback should complement rather than replace mechanisms already employed by amputees, such as visually monitoring the prosthesis, sensing vibrations through the socket, or hearing motor sounds.

Reducing prosthesis noise is another frequently mentioned need that leaves room for improvement. Beyond the addition of sensory feedback, noise impacts overall prosthetic functionality and user satisfaction. Sounds generated by electrical systems, cable clicks, or air expulsion within the socket can cause discomfort and embarrassment [34,35,41,48,53]. Enhancing the silent operation of prostheses would significantly contribute to a more positive user experience.

The adoption of advanced technologies, such as motion pattern recognition and gesture commands, is considered a promising approach to facilitating intuitive prosthetic control [33,35,47,56,57]. In this context, artificial intelligence plays a pivotal role in enhancing the interaction between the user and the device [62].

In addition to advances in functionality, ergonomic needs complement the prosthesis’s effectiveness and acceptance. The reduction in prosthetic weight is widely cited [30,33,41,53]. Lighter prostheses, with well-distributed weight positioned closer to the residual limb [38], can significantly enhance the user experience by reducing physical strain and increasing efficiency in daily activities. Weight is intrinsically linked to prosthesis comfort, which is a frequently mentioned need directly associated with prosthesis acceptance and usage duration [32,53]. Issues such as pain, fatigue [58,59], poor fit, friction, excessive sweating, and skin irritation [33,36,39,48,52,59,60] are commonly reported barriers. Developing prostheses that address these challenges with adjustable designs and breathable, comfortable materials is essential for minimizing discomfort and increasing user acceptance. The ease of donning and doffing the prosthesis [32,38,40], alongside proper fit, is also crucial in reducing pain and skin issues [32,33,48]. Customizing the socket is presented as a practical solution to improve comfort, reduce the need for frequent repairs, and optimize user adaptation [34].

Regarding aesthetics, L. Jabban et al. [53] compare the prosthesis’s appearance to clothing, suggesting that using a prosthesis should be akin to wearing a garment chosen to feel “nice.” This comparison highlights the role of aesthetics in self-expression, identity, and psychological comfort. The desired aesthetics tend to vary across ages and personalities [34,37]. While not uniform [33,38], it is generally observed that children tend to prefer more colorful designs that draw attention [39], whereas adults lean toward natural appearances that match their skin tone [30,34,39,40,41] and can be camouflaged among clothing without causing damage [36,53]. Many users prefer to go unnoticed and avoid unwanted attention [53,59] revealing a strong link between aesthetic choices and the psychological need to minimize stigma and social exposure. Strategies such as hiding the prosthesis in a pocket or covering it with dressings, scarves, or long sleeves [38,42,51,53] reveal a tendency for wearable prostheses, where ergonomic integration with clothing supports both physical comfort and psychological well-being. This underscores the importance of balancing functionality and aesthetics to align with user preferences.

Based on the numerous instances in which dissatisfaction with aesthetics is reported [30,33,34,41,42,47,63] and the strategies adopted by users, it is evident that commercial prostheses still do not fully meet users’ aesthetic needs. Such dissatisfaction can negatively affect self-esteem, body image, and long-term prosthesis acceptance. However, Davidson et al. [51] suggest that using prostheses may eliminate the need to cover up limb loss. Despite varying aesthetic preferences, there is a unanimous preference for anatomically accurate hands and proportional fingers [38,39,41] which supports psychological needs related to body integrity and identity reconstruction. These findings align with the importance of customization [33,38], previously noted regarding functional and ergonomic needs, as customization also contributes to psychological comfort by enhancing confidence and perceived control over the device, and the relevance of defining target groups during prosthesis development [37,38]. Some studies indicate that prosthesis choice and usage also vary depending on the level of amputation, gender, and age group [36].

Intricately linked to the aforementioned needs (ergonomic and functional) are psychological needs related to confidence, trust, and perceived safety when using the prosthesis. Prosthesis robustness and the sense of security [38,40,53,58] are frequently reported as essential requirements, as they directly influence users’ confidence and willingness to rely on the device during daily activities, as they directly influence users’ confidence and willingness to rely on the device during daily activities. Users often highlight issues with prosthesis stability and movement, particularly in situations demanding quick or forceful actions [38,40,48], which can lead to anxiety, fear of device failure, and reduced prosthesis use. This underscores the importance of designing prostheses that ensure structural and operational reliability across different environments to support both physical performance and psychological well-being. Furthermore, the appearance and functionality of prostheses have a direct impact on amputees’ psychological well-being, influencing the acceptance and adoption of these devices. Acceptance is intrinsically tied to the prosthesis’s ability to meet functional, aesthetic, and ergonomic needs while fostering confidence and a sense of identity. The possibility of customization, both in design and functionality, contributes to promoting embodiment [45], identity [33], and self-confidence, which, in turn, enhances meta-perception [58]. Studies indicate that perceiving the prosthesis as a tool, rather than as an extension of the body, can hinder acceptance [38,53]. This lack of embodiment negatively affects users’ confidence and perceived efficacy in using the device [40,42,53,61]. Therefore, developing prostheses that encourage a more natural integration with the body is essential to overcoming these barriers and increasing their sense of utility and naturalness.

Personalization is a recurring theme in the analyzed studies, encompassing not only aesthetic aspects [30,44,47], but also individual customization [33,35,40,53]. It arises from the need to tailor the device to the user’s personality and visual preferences through aesthetic and design improvements [30,34]. Socket customization, for example, is widely identified as a necessity, both to enhance comfort and to reduce the frequency of adjustments and repairs [30,34,38,40,51,56]. For children, personalization is particularly significant, offering psychological and academic benefits [44]. Customized assistive devices facilitate participation, and given their constant growth, prostheses need to be quickly adjustable or replaceable [39].

Other critical requirements to consider in prosthetic design and aesthetics, in addition to weight and comfort, include price [32,33,35,37,38], accessibility [14,33,37], durability [33,39,48,53], ease and speed of replacement/repair [34,38], water resistance for cleaning and hygiene [38,40], and touch compatibility [35,45]. Beyond the high costs [32,37], the waiting time to obtain, repair, or replace a prosthesis [38,48] is a common concern. Thus, the use of durable, low-cost materials that provide both accessibility and resilience, alongside the adoption of a modular design [40], allowing for part replacement or better fitting techniques, is crucial. Emerging technologies such as 3D printing and soft robotics are considered promising alternatives to reduce costs, increase accessibility, and facilitate prosthesis customization [35,39,40]. Additive manufacturing techniques allow for the rapid and cost-effective fabrication of personalized components, enabling precise adaptation to the anatomical characteristics and functional requirements of individual users. In parallel, soft robotics introduces compliant materials and bio-inspired structures that enhance comfort, safety, and adaptability during use. Together, these technologies support the development of lighter, more durable, and adjustable prosthetic systems, thereby improving ergonomics, user acceptance, and overall functionality while better addressing individual user needs.

It was noted that prosthetic development varies significantly across different age groups and types of amputation. Children, for instance, have distinct needs compared to young people and adults, requiring prostheses that adapt to their growth while offering adequate comfort and functionality [39]. Similarly, congenital amputees may have unique preferences and challenges in using prostheses. While some studies report that congenital children find prostheses practical for various activities [44], others suggest that these devices may be perceived as obstacles, warranting further investigation [53]. The analyzed sample, predominantly composed of acquired amputees, seems to reflect mainly the needs of this specific group. However, future studies need to explore the particularities of other user profiles to ensure prosthetic development meets diverse needs effectively.

Despite recent advances, the literature continues to reveal persistent and unresolved challenges in upper limb prosthesis use that directly affect long-term acceptance and abandonment. Recent evidence confirms that usability-related factors such as comfort, weight, control, and reliability are strongly associated with prosthesis adoption and usage [14,64], yet these factors remain recurrent sources of dissatisfaction across user populations. Even when prostheses are worn and initially accepted, as shown in transradial contexts by Pickard et al. [65], issues related to heat discomfort, maintenance, durability, and long-term support persist, threatening sustained use over time. Moreover, functional limitations continue to extend beyond task performance, influencing users’ confidence, embodiment, and social participation, thereby reinforcing the interdependence between usability, functionality, and psychosocial outcomes. Taken together, these findings indicate that the core problem is not a lack of evidence identifying critical factors, but the continued absence of solutions that effectively reconcile these interrelated needs in a way that supports intuitive use, psychological well-being, and long-term prosthesis acceptance. Addressing this unresolved interdependence remains essential to reducing abandonment and improving user-centered prosthetic outcomes.

In summary, the findings suggest that the prosthesis development system should acknowledge and prioritize user needs over time, considering the changes that occur at different stages of life and during adaptation to the device. Integrating emerging technologies and user-centered design methodologies could lead to more satisfactory and functionally appropriate solutions. To improve current prostheses, it is recommended to develop personalized solutions with custom sockets and design concepts that merge aesthetics with individual functionality. The reorganization of internal components [38], using emerging technologies such as 3D printing and soft robotics, could better meet the specific needs of each user. However, these studies emphasize that technology alone does not solve all challenges [35,37,39,40].

**Figure 7:**
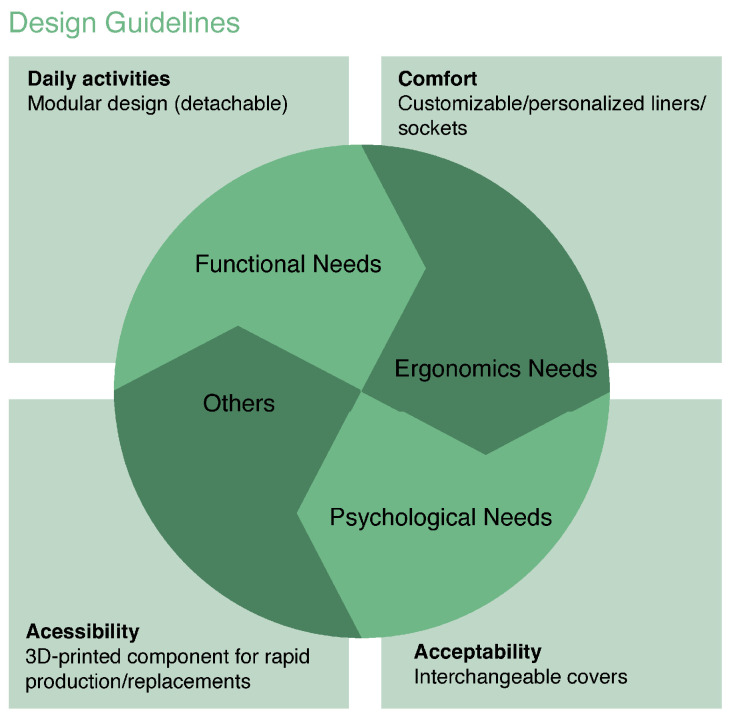
Contributions from the conducted review, including the design guidelines for future works.

The design guidelines presented in Figure 7 were derived through a synthesis of the user-reported needs identified in Table 1. Specifically, recurrent ergonomic, functional, and psychological needs reported across the reviewed studies were grouped and translated into higher-level design principles, ensuring that each guideline directly reflects one or more categories of unmet user needs. This mapping aims to bridge empirical user evidence and actionable design recommendations for prosthetic development [66].

For this reason, adopting design methods involving multidisciplinary teams [30,33,39,41,43] that place users at the center of the process [30,32,43] is highly recommended. These methods help address practical issues and incorporate users’ specific desires, promoting more effective and well-accepted outcomes. Moreover, multidisciplinary teams, involving collaboration between professionals from diverse fields such as engineering, healthcare, design, and psychology, can contribute to innovative solutions by leveraging insights from each domain. This integrated approach aligns with modern trends in medical device design, emphasizing inclusion and interdisciplinarity. When developing new devices, it is crucial to allow customization to meet individual or group-specific needs. Prostheses should also evolve to accommodate changes in users’ needs over time. An adaptable system that allows adjustments and updates as user circumstances change can significantly enhance the user experience and increase adherence to the device. Ultimately, translating user needs into design guidelines that support balanced design solutions across ergonomics, functionality, aesthetics, and financial accessibility is essential for the successful development of prosthetic devices. This balance should take into account not only intrinsic user factors such as age, education, and socioeconomic status but also the context in which the device will be used, including local infrastructure and available technical support.

It is important to note that the findings synthesized in this review primarily reflect the experiences and needs of users of conventional upper limb prostheses. Studies involving advanced surgical interfaces were intentionally excluded to maintain a focus on non-invasive, widely accessible prosthetic solutions. As a result, the conclusions and design guidelines derived from this review should be interpreted within this context and may not be directly generalizable to users of surgically integrated or implant-based prosthetic systems. Future research integrating user-reported evidence from these advanced interfaces may further expand and refine the proposed design considerations.

## 6. Conclusions

This review provides an updated synthesis of user-reported needs for upper-limb prosthetic devices, highlighting functional, ergonomic, psychological, and contextual considerations. By integrating these insights, the study identifies key factors that influence device acceptance, usability, and long-term adherence. Limitations of current prosthetic solutions, including variability in individual needs and gaps in personalization, are discussed. The findings underscore the importance of user-centered design, customization, and interdisciplinary collaboration to develop prostheses that are not only functionally effective but also psychologically and socially supportive.

Given the rapid evolution of this field, future work should include continuous literature updates to integrate emerging evidence and maintain the timeliness of these findings. Furthermore, future research should focus on longitudinal studies and adaptive systems that evolve with users’ changing needs, bridging the gap between technological capabilities and user expectations.

## Figures and Tables

**Table 1:** User needs overview.

	User Needs	References	Sample Size
Ergonomic Needs	Comfort (i.e., easy to fit/detach, sweating, chafing, rubbing…)	[32,33,35,36,38,39,40,41,44,48,51,53,55,56,58,59,61]	1902
	Reduce pain/fatigue	[32,35,36,38,39,44,48,52,54,56,58,59]	1166
	Reduce weight	[30,32,33,34,35,36,37,38,39,40,41,46,48,53,54,55,56,59]	1805
	Security	[33,35,38,40,42,45,48,53,58,59,61]	900
Functional Needs	Daily activities (i.e., eating, cooking, dressing, picking up objects, handling utensils…) Work/School (i.e., income-generating activities, writing, typing, driving, playground games…) Leisure and recreative activities (i.e., leisure, sporting, gardening…)	[30,32,33,35,36,37,38,39,40,41,42,43,44,45,47,48,49,50,51,53,54,55,56,57,58,59,60,61]	4233
	Sensory Feedback	[33,35,38,47,48,53,59]	168
	Better dexterity (i.e., wrist flexion, clumsiness…)	[30,33,35,37,38,40,46,48,51,52,54,55,56,57,60]	1205
	Fine motor skills (i.e., grip fine control, delicacy…)	[33,35,38,39,41,46,53,58,61]	975
	Easy to learn/use (i.e., intuitive)	[35,36,41,47,51,53,61]	1575
	Independence	[38,39,40,48,58,59,60]	158
Psychological Needs	Acceptability (of the device and appearance)	[33,35,38,39,40,41,53,58,59,61]	210
	Design personalization (i.e., natural hand or a superhero’s)	[30,35,37,38,39,40,44,45,59,61]	1600
	Social interactions/Draw lower attention.	[33,38,39,41,42,45,46,53,58,59,60,61]	1002
	Embodiment/Confidence/Better mental health/Safety	[38,40,42,45,53,58,59,61]	2340
	Clinical Services (i.e., Lack of training, appropriate psychological support, and personalizing care of effective rehabilitation)	[32,34,35,36,37,38,40,41,48,50,53,54,59,60]	2915
Other Needs	Reduce Price (accessibility)	[32,33,34,35,37,38,39,40,41,48,51,53]	1459
	Reduce Noise	[33,34,35,41,48,53,59]	593
	Durability, repairs, and replacements (i.e., grew break, unreliable…)	[32,33,34,35,36,37,38,39,40,44,48,51,53]	2287
	Battery life (i.e., operating lifetime)	[33,35,38,56]	110
	Water and weatherproof Ease of cleaning	[38,40,48,55]	523

## Data Availability

No new data were created or analyzed in this study.
